# Preliminary Studies on the Antibacterial Mechanism of a New Plant-Derived Compound, 7-Methoxycoumarin, Against *Ralstonia solanacearum*

**DOI:** 10.3389/fmicb.2021.697911

**Published:** 2021-08-06

**Authors:** Songting Han, Liang Yang, Yao Wang, Yuao Ran, Shili Li, Wei Ding

**Affiliations:** Laboratory of Natural Products Pesticides, College of Plant Protection, Southwest University, Chongqing, China

**Keywords:** coumarins, *R. solanacearum*, antibacterial activity, tobacco bacterial wilt, membrane lysis, 7-methoxycoumarin

## Abstract

*Ralstonia solanacearum* (*R. solanacearum*) is one of the most devastating plant bacterial pathogens and leads to serious economic losses in crops worldwide. In this study, the antibacterial mechanism of 7-methoxycoumarin, a new coumarin antibiotic, was preliminarily investigated by the observation of symptoms and physical and biochemical analyses. The results showed that 7-methoxycoumarin significantly suppressed bacterial growth of *R. solanacearum*, with the minimum inhibitory concentration (MIC) and the minimum bactericidal concentration (MBC) values of 75 and 175 mg/L, respectively. Electron microscopy observations showed that the bacterial cell membrane was destroyed after 7-methoxycoumarin treatment. Biofilm formation of *R. solanacearum* was significantly suppressed by 7-methoxycoumarin at concentrations ranging from 25 to 100 mg/L. Furthermore, virulence-associated genes *epsE*, *hrpG*, and *popA* of *R. solanacearum* were significantly inhibited by 7-methoxycoumarin. The application of 7-methoxycoumarin effectively suppressed tobacco bacterial wilt progress in pot experiments, with relative control efficiencies of 83.61, 68.78, and 58.11% at 6, 8, and 10 days post inoculation, respectively.

## Introduction

Tobacco bacterial wilt is a destructive bacterial soil-borne disease caused by *Ralstonia solanacearum* (*R. solanacearum*) that has an enormous impact on tobacco production and causes large annual economic losses (Swanson et al., 2005; Wu et al., 2015; Jiang et al., 2017). *R. solanacearum* invades host plants through root wounds, grows to high cell densities in the plant host and produces a high mass of extracellular polysaccharides, resulting in hindrance of water transportation in the plant, host wilting and death (Zeid, 2002; Li et al., 2017). Currently, the control methods of bacterial wilt include biological, chemical, cultural and integrated management (Bai et al., 2016; Cai et al., 2018). Pesticides discovered and produced by the agrochemical industry exert powerful effects for disease control management. However, long-term unreasonable use of bactericides leads to serious environmental problems inducing successive development of pesticide-resistant pathogens, disrupting the soil ecological balance, and causing the issue of environmental safety (Fujiwara et al., 2011; Gutiérrez-Barranquero et al., 2013). Developing plant varieties with resistance to bacterial wilt is considered to be the most environmentally friendly, economical, and effective method of disease control. However, many crops with the resistance to bacterial wilt show a low yield and poor quality (Shew et al., 2019). The breeding time of resistant varieties is long, and it is difficult to meet the demand for these crops. Therefore, exploring and developing novel, eco-friendly, and efficient bactericides is important for bacterial wilt control.

Biopesticides are regarded as remarkable alternatives to classic agrochemicals, and have played a crucial role in plant disease control. Renewable plant bioresources contain a variety of plant-derived compounds (PDCs) (Hassan et al., 2009; Li et al., 2014; Cai et al., 2020). Recent studies have proven that these compounds can be used as antibacterial agents to suppress soil borne pathogens and promote plant growth (Paret et al., 2010; Abo-Elyousr et al., 2014). Certain PDCs including DIMBOA, lansiumamide B and protocatechualdehyde, suppress the growth of *R. solanacearum*, resulting in the control of plant bacterial wilt (Li et al., 2014, 2016; Guo et al., 2016). 4-Methoxy-cinnamic acid, benzoic acid and *trans*-4-hydroxycinnamohydroxamic acid are demonstrated to target the type III secretion system and biofilm formation of plant pathogens (Khokhani et al., 2013; Li et al., 2015). Currently, a variety of natural products have been developed as leader compounds to design biopesticides (Yang et al., 2018).

Coumarins are natural secondary metabolites comprised of fused benzene and α-pyrone rings produced through the phenylpropanoid pathway and coumarins accumulate in response to infection by plant pathogens (Zeid, 2002; Sun et al., 2014). Coumarins exhibited strong antibacterial activities against both clinical pathogenic bacteria (such as *Escherichia coli*, *Staphylococcus aureus*, and *Pseudomonas aeruginosa*) and plant pathogens (such as *R. solanacearum*, *Alternaria alternata*, and *Botrytis cinerea*) (Goy et al., 1993; Souza et al., 2005). Studies have reported that coumarin exhibits strong antibacterial activity against *R. solanacearum* by inducing cell membrane lysis (Chen et al., 2016). The antibacterial activity of coumarins is related to the polarity of the oxygen-containing substituents on the benzene ring. When the C-7 position on the benzene ring has a methoxy functional group, or the C-6 and C-8 positions have hydroxyl group substituents, the compound exhibits broad-spectrum antibacterial activity (Kayser and Kolodziej, 1999). Hydroxycoumarins, including umbelliferone, esculetin and daphnetin show strong antibacterial activity against *R. solanacearum* (Yang et al., 2018). However, the inhibitory activity of methoxycoumarins against *R. solanacearum* remains largely unclear.

In this study, we demonstrated the antibacterial activity of 7-methoxycoumarin against *R. solanacearum*. The effects of 7-methoxycoumarin on the ultrastructure of bacterial cells and the leakage of intracellular constituents, as well as the activity of biofilm formation and swimming activity, were examined to understand the mechanism of action of plant-derived coumarins against *R. solanacearum*. Furthermore, the control efficiency of 7-methoxycoumarin on tobacco bacterial wilt was evaluated in pot experiments.

## Materials and Methods

### Bacterial Cultures and Compounds

The *R. solanacearum* (CQPS-1) used in this study was collected by the Laboratory of Natural Products Pesticides and was isolated from an infected tobacco plant in Chongqing, China (Liu et al., 2017). *R. solanacearum* was grown at 30°C on a BG medium (Boucher et al., 1985).

The 7-methoxycoumarin (HPLC ≥ 98%) used in the study was purchased from Shanghai Yuanye Bio-Technology Co., Ltd. (Shanghai, China). This compound was dissolved in dimethyl sulfoxide (DMSO) and prepared at a final concentration of 10 mg/mL. Then, the dissolved compound was added to BG or BG agar medium to prepare compound suspensions of different concentrations, and negative control was treated with the same concentration of DMSO solvent (0.4% final concentration).

### Determination of MIC and MBC

The minimum inhibitory concentration (MIC) and minimum bactericidal concentration (MBC) were determined using the agar dilution method with a series of final concentrations ranging from 25 to 200 mg/L, as previously described with minor modifications (Li et al., 2014). Briefly, overnight-cultured *R. solanacearum* suspension (10^8^ to 10^9^ CFU/mL) was diluted with sterile water to 1 × 10^5^ CFU/mL; then 50 μL of diluted bacterial suspension was spread directly on each antibiotic-containing agar dilution plate. The inoculated culture medium was incubated at 30°C. The MIC was defined as the lowest concentration at which no visible growth of *R. solanacearum* occurred after 48 h of inoculation. The MBC was defined as the lowest concentration at which no visible growth of *R. solanacearum* occurred after 96 h of inoculation. All assays were performed at least in triplicate.

### The Growth Curve of *R. solanacearum*

The growth curve of *R. solanacearum* was investigated as in a previous study with minor modifications (Yang et al., 2017). Briefly, 125 μL of overnight-cultured *R. solanacearum* suspension (OD_600_ = 1.0) was added to 25 mL of BG medium supplemented with 7-methoxycoumarin to generate a final concentration of 10, 25, 50, 75, or 100 mg/L. The control treatment was treated with 100 μL of DMSO. Then, the triangular flask was incubated at 30°C for 24 h. Bacterial density was determined by the optical density (OD) at 600 nm every 2 h. Each treatment was repeated three times.

### Biofilm Formation Analysis

The biofilm formation of *R. solanacearum* was carried out in 96-well polystyrene microtiter plates (Zhang et al., 2014). In short, BG medium and 7-methoxycoumarin were mixed in 5 mL sterilized centrifuge tubes to prepare 5, 10, 25, 50, and 100 mg/L concentration, and then 15 μL of bacterial suspension (OD_600_ = 1.0) cultured overnight was added to 96-well polystyrene microtiter plates and incubated without shanking for 24 h at 30°C. The culture was removed carefully, and plates were washed twice with 200 μL of distilled water. The biofilm was dyed by adding 220 μL of crystal violet (0.1%) and incubation at room temperature for 30 min. After dyeing, the crystal violet was removed, and plates were washed twice with 200 μL of distilled water. After removing the floating color, plates were dried at room temperature for 30 min. Then, 200 μL of 95% ethanol was added to dissolve the crystal violet adsorbed on the biofilm for 30 min, and the absorbance value at 530 nm was determined by a microplate reader, and this represented the biofilm formation of *R. solanacearum* under 7-methoxycoumarin treatment. Each treatment was repeated at least three times.

### Swimming Motility of *R. solanacearum* Under 7-Methoxycoumarin Treatment

The swimming motility of *R. solanacearum* was detected in semisolid medium as described in previous research (Tans-Kersten et al., 2004). 7-methoxycoumarin was added to the semisolid motility medium to final concentrations of 10, 25, 50, 75, and 100 mg/L. The bacterial suspension was diluted with sterile water to OD_600_ = 0.1, and 3 μL of bacterial suspension was dropped onto the plate. The inoculated plate was incubated at 30°C without shaking. The diameter of the white zone around the colony was measured 24 and 48 h after inoculation. Each treatment was repeated three times.

### Transmission Electron Microscopy Analysis

The morphological changes of *R. solanacearum* after 7-methoxycoumarin treatment were further studied by electron microscopy, as in previous research with minor modifications (Chen et al., 2014). *R. solanacearum* (OD_600_ = 1.0) suspension was evenly mixed with 37.5 mg/L 7-methoxycoumarin, and incubated at 30°C for 4–6 h. After centrifuged at 8000 rpm for 5 min, the bacterial pellet was resuspended in 1 mL of sterilized deionized water, washed 3 times, and then fixed with 2.5% glutaraldehyde for 12 h. Then, the bacterial pellet was dehydrated with 30, 50, 70, 90, and 100% ethanol of different gradient series for 15 min, smoked with osmium acid for 3 h and observed directly under a scanning electron microscope. *R. solanacearum* cells were fixed with 1% aqueous OsO4 (Fluka, Los-Angeles, CA, United States) and washed with 0.1 M, pH 7.0 phosphate buffers. Thin sections containing the cells were placed on copper grids and observed under a TEM (FEI, Brno, Czechia).

### RNA Extraction and Quantitative Real-Time RT-PCR

The effect of 7-methoxycoumarin on the expression of virulence-associated genes of *R. solanacearum* was evaluated as previously reported (Wu et al., 2015). Briefly, 250 μL of the overnight cultured *R. solanacearum* suspension (OD_600_ = 1.0) was added to 25 mL of BG medium, mixed with 1/2 MIC (37.5 mg/L) of 7-methoxycoumarin, and incubated at 30°C for 6–7 h. The bacterial cells were collected by centrifugation, and the total RNA of *R. solanacearum* was extracted according to the TRIzol method. After cDNA was synthesized by reverse transcription, the key genes of the type III secretion system (*hrpG* and *popA*), extracellular polysaccharide (*epsE* and *xpsR*), swimming motility (*vsrC*), and chemotaxis (*cheA* and *cheW*) were selected for evaluation of gene expression levels. The primers of the tested genes used in this study were list in Supplementary Table 2 and the housekeeping gene *serC* was used as the control. RT-PCR was performed in a CFX96 Manager (Bio-Rad) using an Sso FastTM EvaGreen^®^ Supermix (Bio-Rand, Hercules, CA, United States).

### Control Efficiency of 7-Methoxycoumarin on Tobacco Bacterial Wilt Under Greenhouse Conditions

The naturalistic soil soak assay was used to evaluate the control efficiency of 7-methoxycoumarin on tobacco bacterial wilt as described in a previous study, with minor modifications (Wu et al., 2015). Briefly, unwounded, 6-week-old tobacco plants (*Nicotiana tabacum. L* Yunyan 87) were irrigated with 15 mL of 7-methoxycoumarin to a final concentration of 25, 50, and 100 mg/g soil. The same volume of 0.4% DMSO was used as a negative control, and as a thiadiazol copper with a final concentration of 100 mg/L was used as a positive control. After irrigation for 24 h, individual plants were inoculated by pouring 10 mL of bacterial suspension into the soil to create a final inoculation density of 1 × 10^7^ CFU/g soil. Inoculated plants were placed in the climate room at 28°C with a 14/10 h light/dark cycle. The symptoms of each plant were scored daily using a disease index scale from 0 to 4 (0, no symptoms appeared; 1, 1–25% of leaves wilted; 2, 26–50% of leaves wilted; 3, 51–75% of leaves wilted; 4 indicated 76–100% of leaves wilted). Individual treatments contained 12 plants for each independent experiment, and the assay was repeated three times. To determine the disease index and the control efficiency, we used the following formulas:

$$\mathrm{Disease}\,\mathrm{index}=\frac{\sum \left(n\,i-v\,i\right)}{N\times 4}\times 100$$

where, *ni* = the number of plants with the respective disease index, *vi* = disease index (0, 1, 2, 3, and 4), and N = the total number of plants used in each treatment.

$$\mathrm{Control}\,\mathrm{efficiency}=\frac{\left(C\,K-T\right)}{C\,K}\times 100$$

where, T = the disease index of treatment, and CK = the disease index of the control group.

### Statistical Analyses

The data were analyzed with Excel 2010 and the SPSS17.0 statistical software program (SPSS Inc, Chicago, IL, United States) using Student’s *t*-test and ANOVA under the significance level of 0.05, 0.01 (*P*-value = 0.05, *P*-value = 0.01).

## Results

### MIC and MBC of 7-Methoxycoumarin Against *R. solanacearum*

**FIGURE 1:**
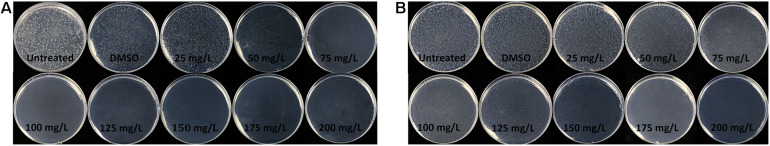
The minimum inhibitory concentration (MIC) and minimal bactericidal concentration (MBC) of 7-methoxycoumarin on *R. solanacearum* was determined using the agar dilution method at the concentrations ranging from 25 to 200 mg/L. **(A)** 48 h after inoculation. **(B)** 96 h after inoculation.

The minimal inhibitory concentration (MIC) and minimum bactericidal concentration (MBC) of the pathogenic bacterium *R. solanacearum* were determined by the solid dilution method. As shown in Figure 1, the MIC and MBC of 7-methoxycoumarin were 75 and 175 mg/L, respectively.

### 7-Methoxycoumarin Inhibits the Growth of *R. solanacearum*

**FIGURE 2:**
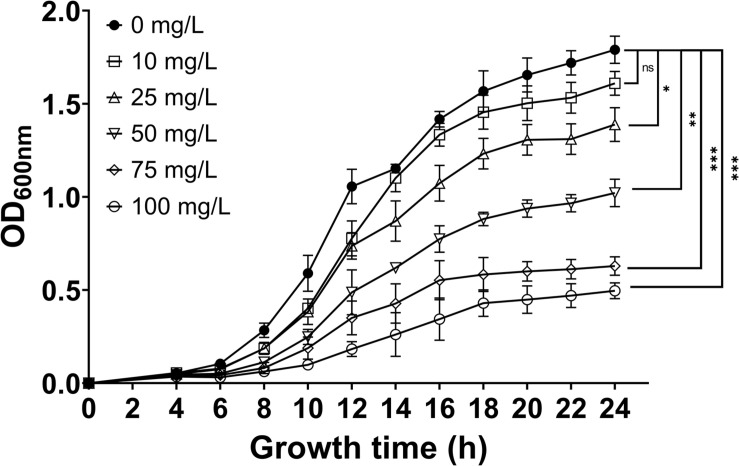
The effect of 7-methoxycoumarin at concentrations ranging from 10 to 100 mg/L on the growth of *R. solanacearum*, (* indicates *p* < 0.05, ** indicates *p* < 0.01, and *** indicates *p* < 0.001).

Further investigation into the inhibitory effect of 7-methoxycoumarin against *R. solanacearum* was performed. As shown in Figure 2, the growth of *R. solanacearum* was significantly inhibited at concentrations of 7-methoxycoumarin ranging from 25 to 100 mg/L and the antibacterial activity of 7-methoxycoumarin against *R. solanacearum* was concentration-dependent. As shown in Supplementary Table 1, the IC_50_ value of 7-methoxycoumarin was 52.98 mg/L after 24 h of culture.

### 7-Methoxycoumarin Reduces the Biofilm Formation of *R. solanacearum*

**FIGURE 3:**
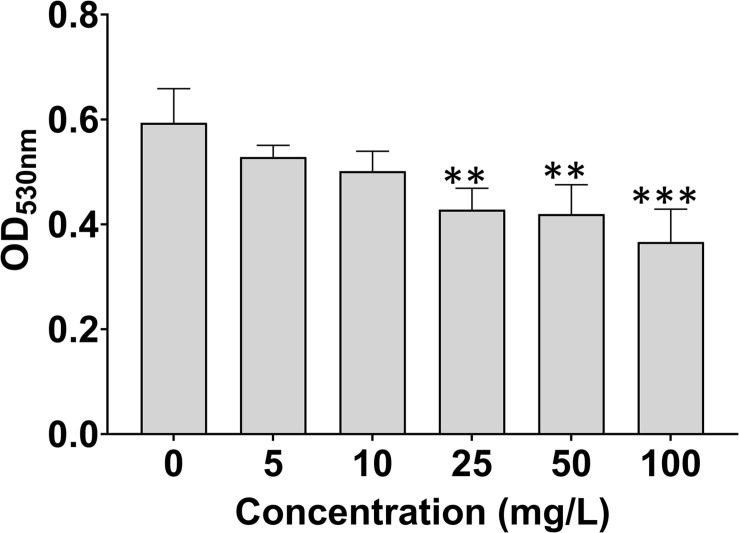
Effects of 7-methoxycoumarin on biofilm formation of *R. solanacearum*. OD530 values were quantified after treatment with different concentrations of 7-methoxycoumarin at 30°C for 24 h in 96-well plates; ** indicates *p* < 0.01 and *** indicates *p* < 0.001).

As shown in Figure 3, 7-methoxycoumarin significantly reduced the biofilm formation of *R. solanacearum*. The biofilm formation treated by DMSO was 0.59, significantly high than *R. solanacearum* supplemented with 25, 50, and 100 mg/L 7-methoxycoumarin, OD_530nm_ were 0.43, 0.42, and 0.37, respectively. Specifically, the inhibitory effects of 7-methoxycoumarin were 21.25, 23.20, and 28.76% at concentrations of 25, 50, and 100 mg/L, respectively (Supplementary Figure 1).

As shown in Supplementary Figure 2, the swimming motility diameter of *R. solanacearum* 24 and 48 h after treatment with 7-methoxycoumarin was not different from that of the control treatment.

### The Effect of 7-Methoxycoumarin on the Cell Morphology of *R. solanacearum*

**FIGURE 4:**
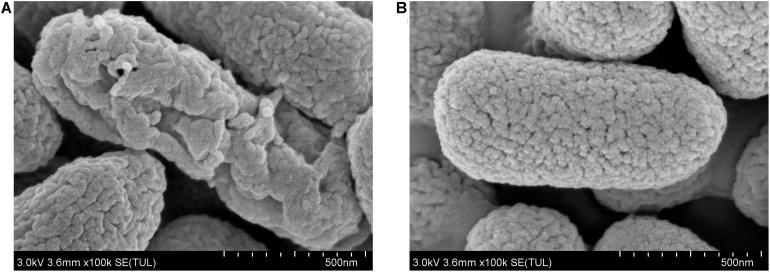
SEM images of *R. solanacearum* cells treated with **(A)** 7-methoxycoumarin; **(B)** DMSO.

**FIGURE 5:**
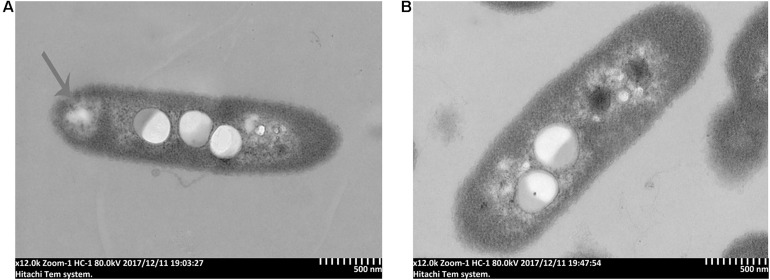
TEM images of *R. solanacearum* cells treated with **(A)** 7-methoxycoumarin; **(B)** DMSO.

To further study the antibacterial mechanism of 7-methoxycoumarin, the morphological changes of *R. solanacearum* after treatment with 7-methoxycoumarin were observed by SEM and TEM. As shown in Figure 4, negative control bacterial cells still maintain their integrity. In contrast, the surfaces of *R. solanacearum* after exposed to 7-methoxycoumarin (37.5 mg/L) were rough and obviously wrinkled. The damaged cell is also observed in TEM image as shown by the gray arrow in Figure 5, it is shown that 7-methoxycoumarin could damage or deconstruct cell walls to penetrate the bacterial cells, this damage may lead to the leakage of lysosomal contents resulting eventually in cell death.

### 7-Methoxycoumarin Suppresses the Expression of Virulence-Associated Genes in *R. solanacearum*

**FIGURE 6:**
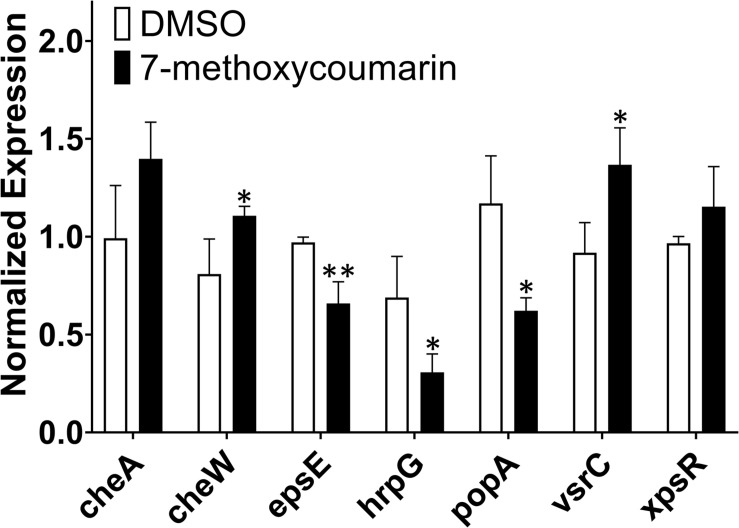
The expression of some virulence-associated genes of *R. solanacearum* was quantified by qRT-PCR after treatment with or without 7-methoxycoumarin (* indicates *p* < 0.05 and ** indicates *p* < 0.01).

In this study, qRT-PCR was used to evaluate the transcriptional expression of the major pathogenic genes of *R. solanacearum* treated with or without 7-methoxycoumarin. As shown in Figure 6, 7-methoxycoumarin inhibited the expression of type III secretion system associated-genes (*popA* and *hrpG*) and the extracellular polysaccharide synthesis gene *epsE*. However, 7-methoxycoumarin was found to induce the expression of *vsrC* and *cheW*. The results indicated that 7-methoxycoumarin can inhibit the genes of the type III secretory system and extracellular polysaccharides, demonstrated that this compound might has a certain preventive effect against host infection by *R. solanacearum*.

### Control Effect of 7-Methoxycoumarin on Tobacco Bacterial Wilt Disease

**FIGURE 7:**
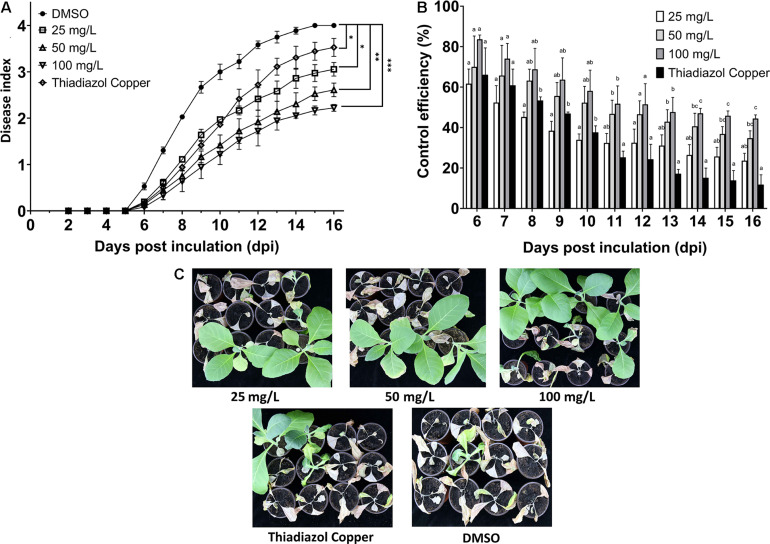
Relative control effects of each treatment on pot experimental tobacco seedlings. **(A)**:The disease index of tobacco wilt treated with 25, 50, and 100 mg/L 7-methoxycoumarin. **(B)** The control effect of 7-methoxycoumarin. **(C)** The control effect after 16 days of inoculation with *R. solanacearum* (* indicates *p* < 0.05, ** indicates *p* < 0.01, and *** indicates *p* < 0.001). Different lowercase letters on the histogram of the same group indicate significant differences between different treatments, *p* < 0.05).

Based on the strong antibacterial activity and biofilm formation inhibition of *R. solanacearum* by 7-methoxycoumarin, we examined the effect of irrigated roots with 7-methoxycoumarin on incidence of tobacco bacterial wilt. As shown in Figure 7A, the disease index of DMSO treatment was 0.53, 2.03, 3.00, and 3.58 at 6, 8, 10, and 12 days, respectively. Compared with DMSO, 7-methoxycoumarin treatments significantly altered the disease index of bacterial wilt and delayed plant wilting. As shown in Figures 7B,C, 7-methoxycoumarin treatment at a concentration of 100 mg/L had control efficiencies of 83.61, 68.78, 58.11, and 51.48% at 6, 8, 10, and 12 days after inoculation, respectively, which significantly higher than the positive control treatment with 100 mg/L thiadiazol copper, with control efficiencies of 66.11, 53.39, 37.61, and 24.37% at 6, 8, 10, and 12 days, respectively.

## Discussion

Traditional chemical pesticides produce environmental pollution and cause damage to the human body during their use (Dasgupta et al., 2007; Acero et al., 2008). Because of their ecofriendly sources and non-toxicity to humans, PCDs have gradually come to be considered a new type of pesticide that can replace traditional chemical pesticides. It has been reported that lansiumamide B, protocatechualdehyde and methyl gallate in PCDs have strong inhibitory effects on *R. solanacearum* to control the occurrence of tobacco bacterial wilt (Yuan et al., 2012; Fan et al., 2014; Wu et al., 2015). Previous studies show that hydroxycoumarins reduce the pathogenicity of *R. solanacearum* by suppressing T3SS and biofilm formation (Yang et al., 2018). However, the inhibitory activity of methoxycoumarins against *R. solanacearum*, especially the mechanism of action, has not been reported.

This article is the first study of the antibacterial effect of 7-methoxycoumarin on *R. solanacearum*, and further elucidates its antibacterial mechanism. According to our results, 7-methoxycoumarin inhibited the growth of *R. solanacearum* in both liquid medium and on solid medium (Figures 1, 2). Previous studies have identified phenyl derivatives as antimicrobial agents for the control of *Pseudomonas aeruginosa*, *Bacilus subtillis* and *Xylella fastidiosa* (Alam, 2004; Maddox et al., 2010). The antibacterial mechanism of phenolic compounds is similar to that of carvacrol and thymol, resulting in a change in membrane potential (Xu et al., 2008). According to these observations, 7-methoxycoumarin caused ruffles and disruption to the cell membrane of *R. solanacearum* (Figures 4, 5). Previous reports indicate that 7-methoxycoumarin has an inhibitory effect on some gram-negative bacteria, including *E. coli, V. cholerae*, and *Pneumococcus* (Céspedes et al., 2006). Therefore, consideration of strong inhibition of *R. solanacearum* and environmental ecofriendly, 7-methoxycoumarin has great potential for application for plant disease control in the future.

As a plant secondary metabolite, coumarins have inhibitory activity against diverse plant diseases, including bacteria, fungi, and viruses (Goy et al., 1993). Several coumarins obtained from different plant species revealed strong antibacterial activity against human associated *E. coli*, *S. aureus*, and *P. aeruginosa* (Khan et al., 2010; Gnonlonfin et al., 2012). The young leaves of *N. attenuata* exhibited an increased resistance to *A. alternata*, which is associated with scopoletin accumulation (Sun et al., 2014). When Arabidopsis was infected by *Pythium sylvaticum*, scopolamine was utilized to synthesize a huge number of inhibitory substances-scopoletin, which concentrated at sites of infestation to inhibit the normal growth of *Phytophthora* (Bednarek et al., 2005). Recently, it was proven that scopoletin induces the accumulation of reactive oxygen species (ROS) such as H_2_O_2_ and O^2–^, which prevents Asian soybean rust from infecting Arabidopsis (Beyer et al., 2019). Coumarins might act as plant antibacterial agents or immunity elicitors, which plays an important role in plant defense.

Like many plant pathogens, *R. solanacearum* forms biofilm-like aggregates in the roots of host plants, leading to bacterial invasion and infection (Yao and Allen, 2007). Studies have found that coumarins, such as scopoletin, umbelliferone, daphnetin, and esculetin, are secreted by plants to protect themselves from the attack of pathogens (Kai et al., 2006). In this study, 7-methoxycoumarin significantly inhibited the biofilm formation of *R. solanacearum*, but had no significant effect on the swimming motility of *R. solanacearum* (Figure 3 and Supplementary Figure 1). We speculated that 7-methoxycoumarin is more likely to reduce host infection by *R. solanacearum* via affecting biofilm formation. Nevertheless, further elucidation of this mode of action may need a more systematic study of the relationship between 7-methoxycoumarin, *R. solanacearum*, and host plants.

Genes related to *R. solanacearum* pathogenicity have been comprehensively and thoroughly studied. For example, type III secretion system genes *hrpG* and *popA* are involved in the regulation of bacterial infection by *R. solanacearum* (Belbahri et al., 2001; Liu et al., 2014). The results of this study showed that 7-methoxycoumarin significantly inhibited the expression of *hrpG* and *popA*, but had little effect on the expression of *cheA* and *xpsR*. These results indicated 7-methoxycoumarin might suppress type III secretion system and biofilm formation of *R. solanacearum*. Further studies should be explained the effect of 7-methoxycoumarin on *popA*.

The control effect of the 100 mg/L 7-methoxycoumarin treatment on tobacco bacterial wilt was not significantly different from that of the control agent thiadiazol copper in the early stage of the disease, but the lasting time was better than that of thiadiazol copper, and the control effect in the later stage of the disease could still reach to 44.44%. Although 7-methoxycoumarin has shown potential as an effective plant-derived antibacterial agent against bacterial wilt, it is not clear whether this effect can be sustained in different field environments.

In conclusion, 7-methoxycoumarin is a potential antibacterial agent in plants, which is more likely to act as a crucial remedy for future field applications for bacterial wilt. Exposure to 7-methoxycoumarin could protect tobacco plants against *R. solanacearum* by strongly inhibiting biofilm formation, and killing the bacteria. Therefore, our study provides environmentally friendly and effective tactics for the research and development of tobacco bacterial wilt control agents and might be extended to the application of other plant disease control in the future.

## Conclusion

In summary, a new plant-derived compound, 7-methoxycoumarin, exhibited strong antibacterial activity against *R. solanacearum*. 7-Methoxycoumarin significantly inhibited biofilm formation and induced bacterial cell membrane lysis. Virulence-associated genes *epsE*, *hrpG*, and *popA* were significantly suppressed by 7-methoxycoumarin. Finally, the new antibacterial agent 7-methoxycoumarin suppressed tobacco bacterial wilt progress and lead to better control effect on tobacco bacterial wilt. This study suggests that 7-methoxycoumarin has potential application in the control of plant bacterial wilt and other plant diseases in the future.

## Data Availability Statement

The raw data supporting the conclusions of this article will be made available by the authors, without undue reservation.

## Author Contributions

WD and LY conceived and designed the experiments. SH, LY, YW, and YR performed the experiments. SH, LY, and SL analyzed the data. SH, WD, and LY wrote the article. All authors contributed to the article and approved the submitted version.

## Conflict of Interest

The authors declare that the research was conducted in the absence of any commercial or financial relationships that could be construed as a potential conflict of interest.

## Publisher’s Note

All claims expressed in this article are solely those of the authors and do not necessarily represent those of their affiliated organizations, or those of the publisher, the editors and the reviewers. Any product that may be evaluated in this article, or claim that may be made by its manufacturer, is not guaranteed or endorsed by the publisher.
