# Supplementary material for: Preliminary Studies on the Antibacterial Mechanism of a New Plant-Derived Compound, 7-Methoxycoumarin, Against Ralstonia solanacearum
Source: Front Microbiol. 2021 Aug 6;12:697911. doi: 10.3389/fmicb.2021.697911 (PMC8377673; doi:10.3389/fmicb.2021.697911)
Supplement: Supplementary file 1 [file Data_Sheet_1.docx]

Supplementary Material

**Preliminary studies on the antibacterial mechanism of a new plant-derived compound, 7-methoxycoumarin, against *Ralstonia solanacearum***

Songting Han^#^, Liang Yang^#^, Yao Wang, Yuao Ran, Shili Li, Wei Ding*

Laboratory of Natural Products Pesticides, College of Plant Protection, Southwest University, Chongqing 400715, China

*Correspondence:

Wei Ding. Laboratory of Natural Products Pesticides, College of Plant Protection, Southwest University, 400716 Beibei Chongqing, China. E-mail: dwing@swu.edu.cn; Tel. /Fax: +86-23-6825-0953

**Table. S1**

**Table S1 The IC_50_s of 7-methoxycoumarin against *R. solanacearum***

| 7-methoxycoumarin |  | Toxicity Regression equations | IC_50_ (mg/L) | R Value |
| --- | --- | --- | --- | --- |
|  | 12 h | Y= 1.534X + 2.617 | 35.74 | 0.8785 |
|  | 24 h | Y= 1.913X + 1.701 | 52.98 | 0.9757 |

**Table. S2**

**Table S2 . Primers used in this study**

| Name | Sequence (5′ to 3′) | Source |
| --- | --- | --- |
| *serc-F* | CCCACCTACGCCATCTATGT | (Wu, Ding, Zhang, Liu, & Yang, 2015) |
| *serc-R* | TTGAGGAAGAACGGCACATT |  |
| *cheA-F* | GCTTCGCCACCATGTATTCG | (Li et al., 2016) |
| *cheA-R* | CTGAACGCCATCTTCCGTTG |  |
| *cheW-F* | GACGACGGTGTACTGGTTGT | (Li et al., 2016) |
| *cheW-R* | AAACACGCGATCGGTGAAGA |  |
| *epsE-F* | CTGGATAAAGCCACGCAAAG | (Wu et al., 2015) |
| *epsE-R* | CAGTGGTACATCGCCATCAC |  |
| *hrpG-F* | GTCTTCACGGTCTGCGAACT | (Wu et al., 2015) |
| *hrpG-R* | ATTGACCTCCAATCCATCCA |  |
| *popA-F* | AGGATCTGCTCGATCTCCTG | (Wu et al., 2015) |
| *popA-R* | AAGACCTGGTGAAGCTGCTG |  |
| *vsrC-F* | ACCACCCTCTCGCCTTATCT | (Yang et al., 2016) |
| *vsrC-R* | ACAGCCAGACATCCAGCAG |  |
| *xpsR-F* | AGATCGACATAGCGCTGCTT | (Wu et al., 2015) |
| *xpsR-R* | TTACTTTGCGGACCTGCTCT |  |

**Fig. S1**


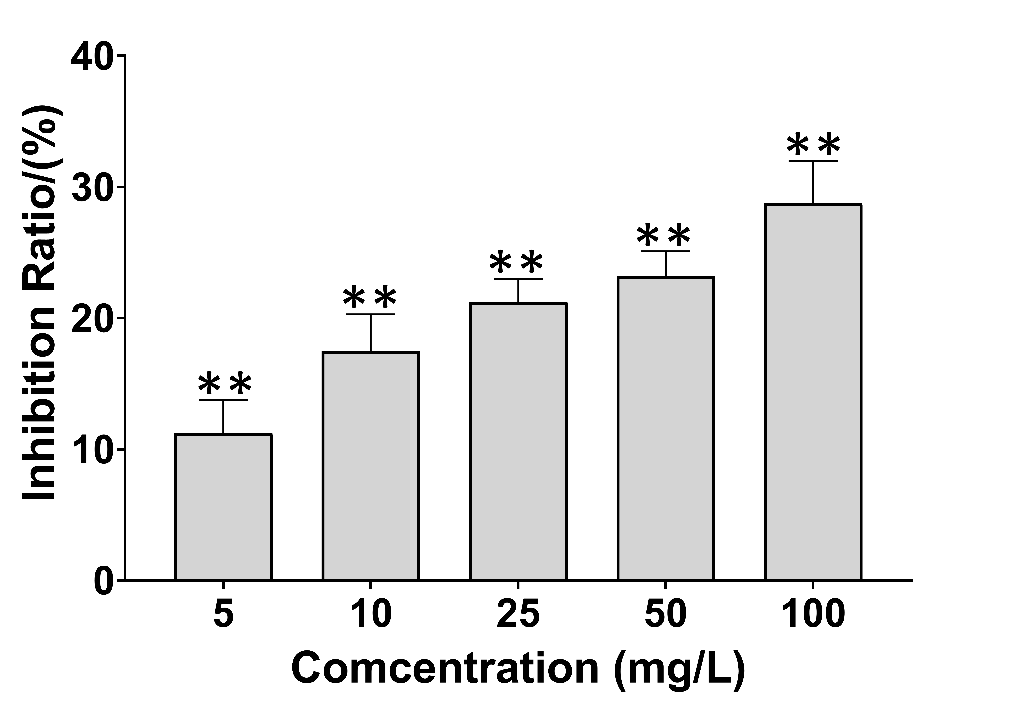


**Fig. S1. Effects of 7-methoxycoumarin on biofilm formation of *R. solanacearum*. Biofilm inhibition (%) was quantified after treatment with different concentrations of 7-methoxycoumarin at 30 ℃ for 24 h in 96-well plates** **(* indicates p < 0.05, ** indicates p < 0.01 , *** indicates p < 0.001).**

**Fig. S2**


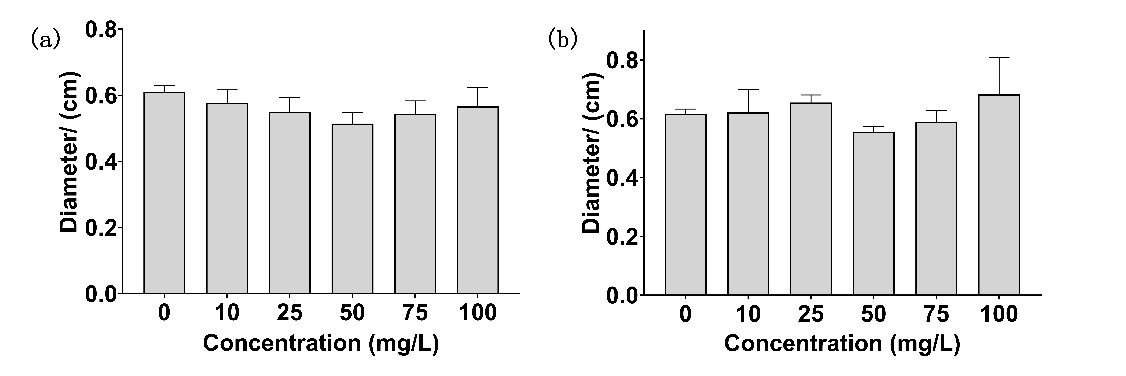


**Fig. S2. Motility of *R. solanacearum* after treatment with 7-methoxycoumarin. (a) The swim diameter of *R. solanacearum* after 24 h of treatment; (b) The swim diameter of *R. solanacearum* after 48 h (* indicates p < 0.05, ** indicates p < 0.01 , *** indicates p < 0.001).**

**References**

Li, S., Yu, Y., Chen, J., Guo, B., Yang, L., and Ding, W. (2016). Evaluation of the Antibacterial Effects and Mechanism of Action of Protocatechualdehyde against *Ralstonia solanacearum*. *Molecules,* 21(6), 754. doi:10.3390/molecules21060754

Wu, D., Ding, W., Zhang, Y., Liu, X., and Yang, L. (2015). Oleanolic acid induces the type III secretion system of *Ralstonia solanacearum*. *Frontiers in microbiology,* 6, 1466. doi:10.3389/fmicb.2015.01466

Yang, L., Ding, W., Xu, Y., Wu, D., Li, S., Chen, J., and Guo, B. (2016). New Insights into the Antibacterial Activity of Hydroxycoumarins against *Ralstonia solanacearum*. *Molecules,* 21(4), 468. doi:10.3390/molecules21040468
